# Stillbirth – a challenge for the 21st century

**DOI:** 10.1186/s12884-016-1181-8

**Published:** 2016-12-10

**Authors:** Alexander E. P. Heazell

**Affiliations:** 1 St. Mary’s Hospital, Central Manchester University Hospitals NHS Foundation Trust, Manchester Academic Health Science Centre, Manchester, M13 9WL UK; 2 Maternal and Fetal Health Research Centre, School of Medical Sciences, Faculty of Biological, Medical and Human Sciences, University of Manchester, 5th floor (Research), St Mary’s Hospital, Oxford Road, Manchester, M13 9WL UK

There are an estimated 2.6 million stillbirths each year [1]. This significant loss of life is frequently unrecognized and hidden from view due to the stigma associated with stillbirth in many countries [2]. Consequently, stillbirth does not receive the prominence it needs in order to influence international policy [3]. Earlier this year these issues were presented in the Ending Preventable Stillbirths Series in the Lancet [1–5]. This outlined ongoing challenges preventing stillbirths from being addressed. This series of papers in BMC Pregnancy and Childbirth develops the work presented in the Ending Preventable Stillbirths Series. These papers are a cogent reminder of the impact of stillbirth and provide considerations for how this might be addressed.

Firstly, they remind us that stillbirth is a global issue; 98% of stillbirths occur in low and middle income countries (LMICs) [1]. Despite this knowledge, challenges remain in data collection which prevent understanding of the number of stillbirths and their underlying causes. To address issues with data collection e-Health and m-Health strategies have been proposed, but the potential of these approaches has yet to be realized [6]. Data also need to reflect the global nature of stillbirth but to date there is no globally acceptable classification system for perinatal deaths [7, 8]. Studies presented here outline the key characteristics required for such a system including the capacity to accumulate data from both high income and low and middle income settings and for the system to be accessible by e-Health and m- Health [9]. However, of all the classification systems available none had all of the characteristics and most had fewer than half of these facets [7]. It is hoped that the development of the ICD-PM classification will address some of these challenges [10, 11], but this system must evolve and be refined in an ongoing process. Critically, without meaningful data stillbirths will continue to be overlooked and efforts to reduce stillbirth hampered.

Secondly, these papers present the human impact behind the statistics, the negative psychological responses which may clash with societal and cultural expectations of bereaved parents and may result in disenfranchised grief [12]. The stigma of having a stillborn child, particularly in high-burden LMICs prevents parents from being able to acknowledge their child through normal rituals. The human cost extends beyond parents to include the wider family although the consequences vary between cultures and the relationship with the child [13]. Importantly, not all responses were universally negative. Some parents reported a changed approach to their life, self-esteem and identity. The results of the systematic reviews demonstrate that staff behaviour is critical in mediating parents’ responses [14]. Distress may be caused by ritualistic adherence to guidelines rather than the provision of authentic, empathic, respectful care. There are many barriers which must be overcome to deliver high-quality respectful care, including emotional, informational and system factors. It is imperative that international professional organisations such as International Federation of Gynecology and Obstetrics (FIGO) and international confederation of midwives (ICM) give global leadership to address stillbirth and provide optimum care for bereaved parents.

Lastly, it is clear that stillbirth cannot be addressed by healthcare strategies alone. Stillbirth is undoubtedly associated with social and economic determinants of health. Zeitlin et al. demonstrate that women with lower educational attainment and manual workers have a higher stillbirth rate [14]. These issues are widespread across many countries and echo the findings of increased stillbirth rate in socially marginalized populations such as black or minority ethnic groups, indigenous populations and migrants [5]. This provides a clear challenge to governments that initiatives to reduce stillbirth cannot be confined to healthcare interventions. It is essential to keep in mind that investment in care to reduce stillbirth will also reduce maternal and neonatal deaths and infant morbidity, providing a quadruple return on investment [15].

If research findings are not connected to initiatives to improve care, the opportunity to prevent stillbirths will be reduced. Research must inform initiatives to develop care such as those outlined here to collect data on stillbirths that can be compared between populations. Research can also develop care for women and their families to address their experiences. Key to this is empathic, respectful care that provides tangible support and information for parents and their families. The greatest challenge is to overcome stigma and taboo that keeps stillbirth hidden. The publication of this series of papers unashamedly attempts to maintain the place of stillbirth in the international agenda.
